# Effect for Human Genomic Variation During the BMP4-Induced Conversion From Pluripotent Stem Cells to Trophoblast

**DOI:** 10.3389/fgene.2020.00230

**Published:** 2020-04-07

**Authors:** Hai-tao Li, Yajun Liu, Hongde Liu, Xiao Sun

**Affiliations:** ^1^State Key Laboratory of Bioelectronics, School of Biological Science and Medical Engineering, Southeast University, Nanjing, China; ^2^The Second Affiliated Hospital of Zhengzhou University, Zhengzhou, China; ^3^Academy of Medical Sciences of Zhengzhou University Translational Medicine Platform, Zhengzhou University, Zhengzhou, China

**Keywords:** genomic variations, pluripotent stem cells, trophoblast, whole genome sequencing, epigenomic and transcriptomic data

## Abstract

The role of genomic variation in differentiation is currently not well understood. Here, the genomic variations were determined with the whole-genome sequencing for three pairs of pluripotent stem cell lines and their corresponding BMP4-induced trophoblast cell lines. We identified ∼3,500 single nucleotide variations and ∼4,500 indels by comparing the genome sequenced data between the stem cell lines and the matched BMP4-induced trophoblast cell lines and annotated them by integrating the epigenomic and transcriptomic datasets. Relatively, introns enrich more variations. We found ∼45% (42 genes) of the differentially expressed genes in trophoblasts that associate genomic variations. Six variations, located at transcription factor binding sites where H3K4me3 and H3K27ac are enriched in both H1 and H1_BMP4, were identified. The epigenetic status around the genomic variations in H1 was similar to that in H1_BMP4. This means that the variation-associated gene’s expression change can not be attributed to epigenetic alteration. The genes associated with the six variations were upregulated in differentiation. We inferred that during the differentiation, an increased in the expression level of the MEF2C gene is due to a genomic variation in chromosomes 5: 88179358 A > G, which is at a binding site of TFs KLF16, NR2C2, and ZNF740 to MEF2C. Allele G shows a higher affinity to the TFs in the induced cells. The increased expression of MEF2C leads to an increased expression of TF MEF2C’s target genes, subsequently affecting the differentiation. Although genomic variation should not be a dominant factor in differentiation, we believe that genomic variation could indeed play a role in the differentiation from stem cells into trophoblast.

## Introduction

Stem cell differentiation involves a complex but poorly understood biological process. Genetic and epigenetic factors have in the past been intensively studied for this process. Some key transcription factors (TFs) affecting differentiation have been identified, such as POU5F1, SOX2, and KLF4 (Nichols et al., 1998; Takahashi and Yamanaka, 2006; Goolam et al., 2016). Previously, our lab identified TFs for DNase I hypersensitive sites based on public available chromatin accessibility data of human embryonic stem cells (ESCs) and BMP4-induced trophoblast cell lines (Liu et al., 2017). The chromatin structure, including nucleosome positions, DNA methylation, and histone modifications also changed during the differentiation (Su et al., 2012; Xie et al., 2013; de Boni et al., 2018). However, genomic variation, another important factor, was rarely considered in the differentiation-related study.

The cells forming the outer layer of a blastocyst in early development are referred to as trophoblasts. These cells significantly contribute to the placenta and membrane, and also provides nutrients to the embryo. This layer of trophoblasts is collectively named “the trophoblast.” The trophoblast is the first cell type differentiated from the zygote during the first stage of pregnancy. Human ESCs provide a very useful model for studying the early development of human embryos and trophoblasts. However, the genomic variation that occurs in the trophoblast differentiation is not well studied *in vivo*. Since Xu et al. proved that bone morphogenetic protein 4 (BMP4) could induce human ESCs, efficiently differentiating to trophoblast lineage, multiple research institutes have adopted this system as a model to study trophoblast lineage specification *in vitro* (Xu et al., 2002). Roberts’s et al. considered this model reliability by analyzing gene expression profiling through RNA sequencing (RNA-Seq) technology (Yabe et al., 2016; Liu et al., 2017).

Recently, studies have demonstrated how genetic variation affects gene expression (Banovich et al., 2018; Delahaye et al., 2018). Kilpinen et al. (2013) suggest that genetic variation appears to be the primary driver of gene expression variation. Furthermore, DeBoever et al. (2017) show that the vast majority of genetic variations associated with gene expression levels are located in the regulatory regions of human induced pluripotent stem cells (iPSCs).

An integrative analysis using combinations of genomic, epigenomic, and transcriptomic data will provide a basis for biomarker discovery and help to provide insight of disease etiology. Genomic sequence variation, epigenetic factor, and gene expression are interdependent and jointly contribute to the normal functioning or dysfunction of tissue (Delahaye et al., 2018). For example, the sequencing variations can alter the TF binding strength to regulate gene expression directly (Karczewski et al., 2011; Madsen et al., 2018; Johnston et al., 2019). We are interested in the role of the genome variations in differentiation from human ESCs to trophoblasts.

In this study, we analyzed the genomic DNA sequences for three paired human ESCs and iPSC (H1, H9, and MRucR) (Sheridan et al., 2019). We demonstrated that some of the genomic variations can affect the differentiation by altering TFs’ binding affinity.

## Materials and Methods

### Whole Genome Sequence of Cell Lines in This Study

Genomic DNA sequences were determined for three stem cells and their corresponding BMP4-induced cell lines (Supplementary Table S1), in which two types of stem cells were human ESC lines (H1 and H9), and third an iPSC cell line, MRucR. The cell lines that differentiated into the trophoblast were named H1_BMP4, H9_BMP4, and MRucR_BMP4, respectively. The three cell lines and the matched BMP4 induced cell lines was obtained from the Roberts’s laboratory, University of Missouri. For more details of these three pairs of cell lines see Sheridan et al. (2019). MRucR iPSC was established from umbilical cords of babies born to mothers who experienced an early-onset form of preeclampsia during their pregnancies. The BMP4-inducing experiment was performed as previously described (Sheridan et al., 2019). Briefly, the trophoblast stem cells were exposed to BMP4 in combination with signaling inhibitors of ACTIVIN-A (A83-01) and FGF2 (PD173074) (BAP treatment) (Sheridan et al., 2019).

### Library Preparation and Sequencing

The quality of isolated genomic DNA was verified using these three methods: (1) DNA purity and concentration were identified by NanoPhotometer^®^ spectrophotometer (IMPLEN, CA, United States) (OD260/OD280). The Optical Density (OD) value of the qualified sample ranged between 1.8 – 2.0. (2) DNA degradation, and suspected RNA/Protein contamination were verified by electrophoresis on 1% agarose gels. (3) The concentration and purity of DNA samples were further quantified precisely by the Qubit DNA Assay Kit in Qubit^®^2.0 Flurometer (Life Technologies, CA, United States). A total amount of 1 μg DNA per sample was required for library generation.

Paired-end DNA libraries were prepared according to the manufacturer’s instructions (Illumina Truseq Library Construction). First, 1.0 μg Genomic DNA was sheared into an average size of 350 base pair (bp) fragments by Covaris S220 sonicator. Second, the ends of the gDNA fragments were repaired; 3′ ends were adenylated. Both ends of the gDNA fragments were ligated at the 3′ ends with paired-end adaptors (Illumina) with a single ‘T’ base overhang, and purified using AMPure SPRI beads from Agencourt. The size distribution and concentration of the libraries were then determined using Agilent 2100 Bioanalyzer and were qualified by real-time PCR (2 nM), respectively. Lastly, DNA libraries were sequenced on Illumina Hiseq X according to the manufacturer’s instructions for paired-end 150 bp reads.

### Whole Genome Sequencing Data Analysis

The raw image files obtained from the Hi-Seq platform were processed with the Illumina pipeline for base calling and stored as FASTQ format (Raw data). Quality control (QC) was as follows: (1) to filter reads with adapter contamination (>10 bp aligned to the adapter allowing ≤ 10% mismatches). (2) to discard the reads containing more than 10% uncertain nucleotides. (3) to discard the paired reads when a single read has more than 50% low quality nucleotides (Phred quality < 5).

After quality control, the sequenced reads were mapped to the GRCh37 assembly of the human genome by Burrows-Wheeler Aligner (BWA) software using default settings (Li and Durbin, 2009). Subsequently, we used Samtools (Li et al., 2009) and Picard^1^ with default settings to sort reads, remove duplicated reads, and to generate the final bam file. If one or more pair read(s) had multiple mapping positions, the best one was selected. If there were multiple best mapping positions, we randomly picked one.

### Variation Calling and Functional Annotation

**FIGURE 1:**
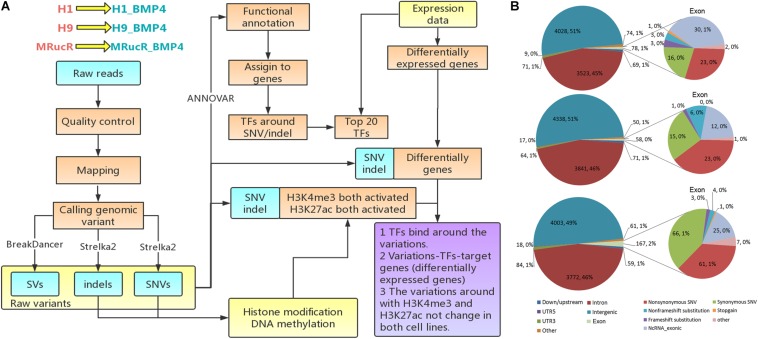
Genomic variations during the differentiation. **(A)** Flow chart of the analysis. **(B)** The variations occurred in different genomic regions. In this figure, down/upstream means the variant overlaps the 1-kb region downstream of transcription end site or upstream of TSS.

The analysis flowchart is shown in Figure 1A. The genomic variations were determined with the whole-genome sequencing for three pairs of pluripotent stem cell lines and their corresponding BMP4-induced trophoblast cell lines and annotated them by integrating the epigenomic and transcriptomic datasets. We only focused on the direct effect on the differentiation of genomic variation which is located in those genomic regions where epigenetic markers remain comparable between H1 and H1_BMP4.

Genomic variations were identified by comparing genomic DNA sequences between stem cell lines and matched BMP4-induced trophoblast cell lines using the following steps. Single nucleotide variation (SNV) and small somatic insertions and deletions (indels) were identified using the Strelka2 (Kim et al., 2018) tool. All ‘PASS’ calls identified by Strelka2 were retained for downstream analyses. BreakDancer (Chen et al., 2009) was applied to detect structural variation (SV). The SVs that received minimal confidence scores (90 for insertions, inversions, deletions, and translocations) were selected for downstream analyses.

The ANNOVAR tool was used to produce statistical analyses of the SNVs/indels (Wang et al., 2010). The variation position, variation type, conservative prediction, and other information were obtained at this step through a variety of databases, including DbSNP, 1000 Genome, and the reference sequence (Sherry et al., 2001; Pruitt et al., 2007; The 1000 Genomes Project Consortium, 2010). The variations were then assigned to genes by associating them with the nearest transcription start sites (TSSs) using the BEDOPS toolkit (Neph et al., 2012) and Gencode v21 human annotation (Harrow et al., 2012).

The regions include exon, intron, intergenic region, UTR5, UTR3, and the upstream (variant overlaps 1-kb region upstream of transcription start site)/downstream (variant overlaps 1-kb region downstream of transcription end site) regions.

The annotation detail of these three cell lines is listed in Supplementary Excel File S1. The version of the bioinformatics tools used is listed in Supplementary Table S2.

### Public Datasets Analyzed in This Study

The goal of the work is to assess the effect of the genomic variations during stem cell differentiation. We therefore need to exclude the effect of the epigenomic variation (Figure 1A). We thus combined DNA methylation, histone modifications, chromatin accessibility, and transcriptomic datasets for the H1 and H1_BMP4 (differentiated with BAP treatment) cell lines. All the ChIP-Seq and DNase-Seq data in H1 and H1_BMP4 were generated by the ENCODE Consortium^2^ and retrieved from the GEO database according to their accession number (Supplementary Table S3) (Lister et al., 2011; Roadmap Epigenomics Consortium et al., 2015; Davis et al., 2018). The sequencing data were mapped to the human reference genome GRCH37/hg19 by BWA. For ChIP-Seq data, we performed peak calling by using the MACS2 tool with default settings (Zhang et al., 2008). Differential peaks between the two cell lines were also identified with MACS2. We are interested in the genomic variations where there is no significant change between the two matched cell lines in the epigenomic data, especially for histone marks H3K27ac and H3K4me3 since the two represent the active state for enhancers and promoters.

The methylation state at CpG sites in whole genome bisulfite sequencing (WGBS) data were mapped to GRCh38/hg38 (Supplementary Table S3) (Roadmap Epigenomics Consortium et al., 2015). We used the liftOver tool to transform the genome coordinates from hg38 to hg19 (Dreszer et al., 2012).

The gene expression data (Xie et al., 2013) were retrieved from the ENCODE project (Supplementary Table S3) with the following identifiers: ENCFF245NXP, ENCFF809MAC, ENCFF051SBM, and ENCFF787HWI. RNA abundance was represented as the logarithm of the transcripts per million (TPM) provided by the RSEM program (Li and Dewey, 2011). The data was used in two aspects. One to identify the differentially expressed genes, so as to check which genes are influenced by the genomic variations. The other is to find the transcription factors that truly have a function in cells. This was done by checking the expression level of the gene that encodes the transcription factor (Figure 1A). Differentially expressed mRNAs were identified with limma (Ritchie et al., 2015). Genes with Fold Change > 2 or <1/2 and false discovery rate (FDR) *p*-value < 0.05 were identified as significantly differentially expressed genes between ESC H1 and differentiated to trophoblast H1_BMP4.

### Prediction for Transcription Factor Binding Sites (TFBSs) Around the Genomic Variations

To estimate the change of the TF binding affinity due to the genomic variation, the 150-bp DNA sequences were extracted around the genomic variations and inputted into a bioinformatics tool, HOMER (Heinz et al., 2010), to calculate the affinity (Figure 1A). Each of the sequences includes one kind of genomic variation. The affinity of a TF to a specific DNA sequence can be estimated by comparing the DNA sequence and the motif of the TF. The motif is the most favorable binding DNA pattern of the TF and can be represented with Position Weight Matrix (PWM). The comparison will be a score on the motif (motif score). A high score means a high affinity between the DNA sequence and the TF. We listed the TFs with a motif score ≥ 10 in the stem cell and its BMP4-induced cells and found the difference of the TFs between the cells.

## Results

### The Distribution of Genomic Variations During the Differentiation

We performed the high-throughput DNA sequencing for three pluripotent stem cell lines (H1, H9, and MRucR) and differentiated trophoblasts (H1_BMP4, H9_BMP4, and MRucR_BMP4). After quality control (Supplementary Figure S1), genomic variations were identified by comparing the DNA sequence between the stem cell lines and matched BMP4-induced trophoblast cell lines. Reads mapping rate was more than 98%, and the sequencing depth was beyond 31X (Supplementary Tables S4, S5). We used Strelka2 to identify SNV and indels. SV was identified with BreakDancer (Chen et al., 2009; Kim et al., 2018). The Flow chart of our analysis is shown in Figure 1A. We identified approximately 3,500 SNVs and 4,500 indels in the ESC and iPSC lines, respectively (Table 1A). The count of SV was relatively rare (∼230) (Table 1A).

**TABLE 1:** Number of genomic variants **(A)** and structural variants **(B)** in which stem cells differentiate into trophoblast cells.

**(A)**					

**Matched hESC-trophoblast cell lines**	**Indels**		**SNV**		**SV**

H1	4071		3781		233
H9	4703		3736		235
MRucR	4729		3435		225

**(B)**					

**Matched hESC-trophoblast cell lines**	**INS**	**INV**	**ITX**	**CTX**	**DEL**

H1	31	2	92	108	0
H9	19	10	96	110	0
MRucR	44	6	84	91	0

*Indels, small somatic insertions and deletions; SNV, single nucleotide variant; SV, structural variation; INS, insertion; INV, inversion; ITX, intra-chromosomal translocation; CTX, inter-chromosomal translocation; DEL, deletions.*

The frequency of SNVs was counted. According to our statistics, the single-base substitution patterns were similar in the three pluripotent stem cells (Supplementary Figure S2a). The highest frequency of SNV was the conversion of cytosine to thymine (C > T), and the lowest frequency of SNV was the conversion of guanine to cytosine (G > C). Among the SVs (Table 1B), the proportion of translocation, including inter- and intra-, was high (about 77%∼87%). The percentage of insertion inversion and deletion was 12% ∼ 22%. We did not detect the structure deletion events here. SV had similar distributions in these three pairs of cell lines (Figure 1B).

**FIGURE 2:**
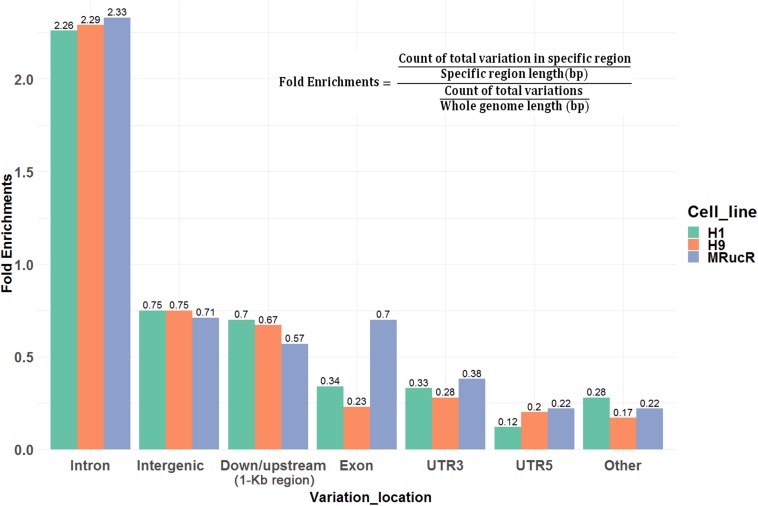
Ratios of the number of variations occurred in certain categories of the genomic regions. Fold enrichments, ratios of the variation counts in a certain category of genomic regions were divided by the proportions of this category in the whole genome length. Down/upstream means the variant overlaps the 1-kb region downstream of the transcription end site or upstream of TSS.

We then annotated SNV and indels with ANNOVAR (Wang et al., 2010). The SNV and indels were counted in different genomic regions (Supplementary Excel File S1). To calculate fold enrichments, ratios of the variation counts in a certain category of genomic regions were divided by the proportions of this category in the whole genome length. The variations were significantly enriched in the introns (Figure 2).

Distribution profiles of SNV/indels in the 2500-bp genomic regions around the TSSs seemly occurred in a periodic pattern in the cell lines (Supplementary Figure S2b). SNP frequency spectra show striking periodicities across nucleosomal regions, and SNPs have a preference for nucleosomes (Langley et al., 2014). We therefore speculated that there is an association between the patterns and nucleosome distribution in this region.

### Transcription Factors That Bind Around Genomic Variations Sites

**FIGURE 3:**
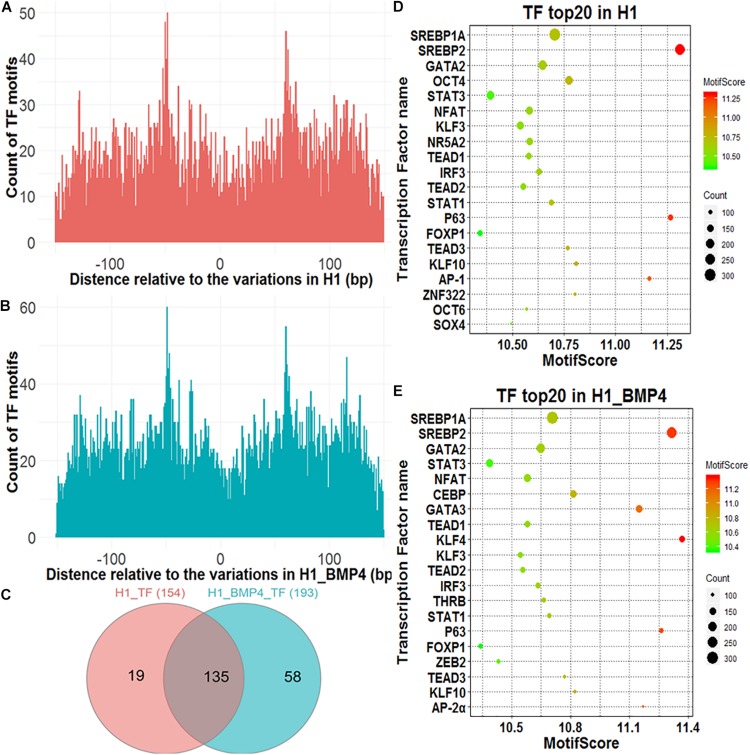
Transcription factors identified around genomic variations sites. **(A)** The Venn diagram describing the overlap between the TFs in H1 and H1_BMP4. **(B,C)** The count of the TF motif around SNV/indels in H1 and H1_BMP4. **(D,E)** The bubble chart of the top 20 key TFs in H1 and H1_BMP4.

A genomic variation may directly alter the affinity of TF’s binding as this variation occurs exactly at TFBSs, thus influencing the transcription levels of the downstream target genes of the TFs. We assessed the effect of the variations on the alteration of TF binding with HOMER (Heinz et al., 2010). The 150-bp DNA sequences around the variation sites were inputted into HOMER to identify motifs of the TFs and compared the affinity of the TFs variation between the stem cell and matched BMP4-induced trophoblast cell lines. In Figures 3A,B, although counts of TF motifs around SNV/indels positions decreased, the number was not zero. There were some motifs that were far away from the SNV/indels. We intend to find out what TFs bind at the SNV/indels-harbored sites.

A TF with its motif in one genomic region does not mean the TF indeed binds at the region in a cell because the TF maybe is not expressed in the cell. We therefore need to check if the TF is abundant enough in the cell, namely, to check if the expression level of the gene encoding the TF is high enough. To do this, a necessary step is to exclude those TFs with a low abundance. The expression of TF-encoding genes was shown for H1 and H1_BMP4 (Supplementary Figure S3). Here, the cutoff of TPM ≥ 3 was used to select the TFs that indeed exist in the cell. We identified 154 and 193 TFs in H1 and H1_BMP4 cells, respectively, with an overlap of 135 TFs (Figure 3C). The top 20 key TFs were chosen by setting TPM ≥ 3 and motif score ≥ 10 (Figures 3D,E and Supplementary Tables S6, S7). In the top 20 TFs of H1 and H1_BMP4, some widely known to be involved in placental development were found, such as GATA3 (Kubaczka et al., 2015), POU5F1 (Wang et al., 2012), and KLF4 (Abad et al., 2013). A target gene of SREBP1A is the transcriptional repressor BHLHB2, which also promotes the differentiation of stem cells to trophoblast giant cells (Lecomte et al., 2010). ZEB2 has recently been identified to play critical roles in the regulation of the epithelial-mesenchymal transition and trophoblast differentiation (DaSilva-Arnold et al., 2019).

We identified the TFs that differ in affinity around the genomic variation sites between H1 and H1_BMP4. The TFs were chosen under conditions of TPM ≥ 3, motif score ≥ 10, and motif score count difference (abs) ≥ 10. We found three TFs (Zfp281, OCTs, and KLF3), whose affinities near the genomic variations differ between H1 and H1_BMP4 cell lines. The three kinds of TFs have a higher motif score count (count of motif score ≥ 10) in H1 than in H1_BMP4. Interestingly, these TFs are widely known to be involved in trophoblast differentiation. For example, Zfp281 (Krüppel-like zinc finger transcription factor), a zinc finger transcription factor, which shapes the transcriptome of trophoblast cells and regulates early placental development, has also been investigated in a recent article (Ishiuchi et al., 2019). Moreover, the other TFs were widely known to be important in placental development (Supplementary Table S8).

In short, we found that there were indeed some genomic variations at TFBSs.

### Correlation Between Genome Variations and Epigenomics

Genome variation was reported to be correlated to epigenetic alteration. Li et al. demonstrated that about 2/3 of eQTLs were due to variations that altered chromatin accessibility or histone marking (Li et al., 2016). We studied the correlation between genetic variations and chromatin alteration in H1 and H1_BMP4 cell lines. The sequencing data of H3K4me3, H3K27ac, H3K27me3, and DNA methylation data were retrieved from ENCODE (Supplementary Table S5).

**FIGURE 4:**
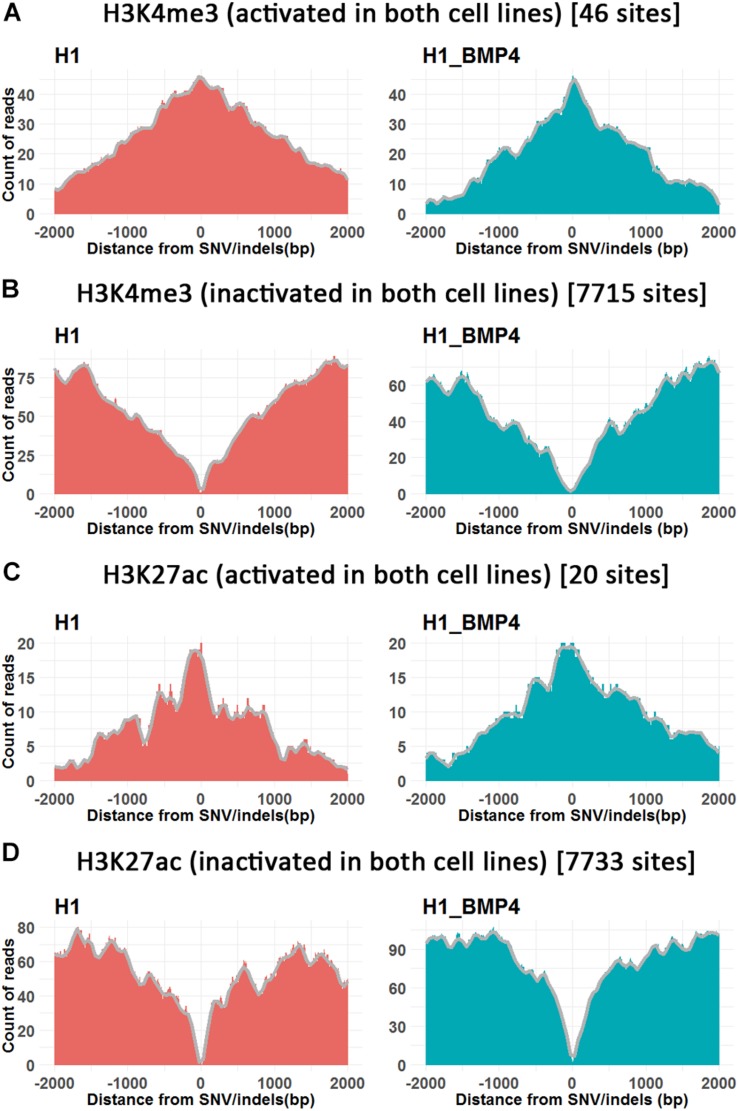
The histone modifications surrounding SNV/indels. *X*-axis is the upstream and downstream 2 Kbp relative to SNV/indels sites. **(A,B)** The number of peak counts on the genomic variations which are located in both promoter activated **(A)** or inactivated **(B)** around H3K4me3 between H1 and H1_BMP4. **(C,D)** The number of peak counts on the genomic variations which are located in both enhancer activated **(C)** or inactivated **(D)** around H3K27ac between H1 and H1_BMP4. The number in parentheses indicates the number of SNV/indels. Complete profiles are shown in Supplementary Figure S4.

In this analysis, the genomic variations were divided into four categories according to whether the variation site was at the peaks of the histone modifications and DNA methylation in H1 and H1_BMP4 (Lister et al., 2011; Roadmap Epigenomics Consortium et al., 2015). We counted the peaks of the histone modifications and DNA methylation within the 4K-bp genomic region around SNV/indel sites (Figure 4 and Supplementary Figure S4).

The enriching regions (peaks) of DNA methylation and H3K27me3 were considered as “inhibited” regions. Modified regions (peaks) by H3K4me3 and H3K27ac indicate “promoter activated” and “enhancer inactivated” regions, respectively. By comparing the epigenomic states of the regions around the genomic variations between the stem cells and matched BMP4-induced trophoblast cell lines, 66 variations that do not associate an epigenomic alteration were identified (Table 2, and Supplementary Excel File S2). Next, we evaluated the effects of the genomic variation around which the epigenetic modifications do not alter between H1 and H1_BMP4.

**TABLE 2:** Number of the genomic variations that occur in regions associating different switches of the epigenomic modifications between H1 and H1_BMP4.

Regions	Modification types	State switch from H1 to H1_BMP4	The number of genomic variations
–	H3K27me3 and DNA methylation	In - > T	0
–	H3K27me3 and DNA methylation	T - > In	0
Enhancer	H3K27ac	I - > A	18
Enhancer	H3K27ac	A - > I	12
Promoter	H3K4me3	I - > A	30
Promoter	H3K4me3	A - > I	30
Promoter	H3K4me3	A - > A	46
Enhancer	H3K27ac	A - > A	20

*In, inhibited; T, transcribed; I, inactivated; A, activated.*

### The Direct Effect of Genomic Variation on Differentiation

We are interested in the gene expression change that is only caused by the genomic variation instead of the epigenetic variation. We therefore only focused on the genomic variations which are located in those genomic regions where epigenetic markers remain comparable between H1 and H1_BMP4.

**FIGURE 5:**
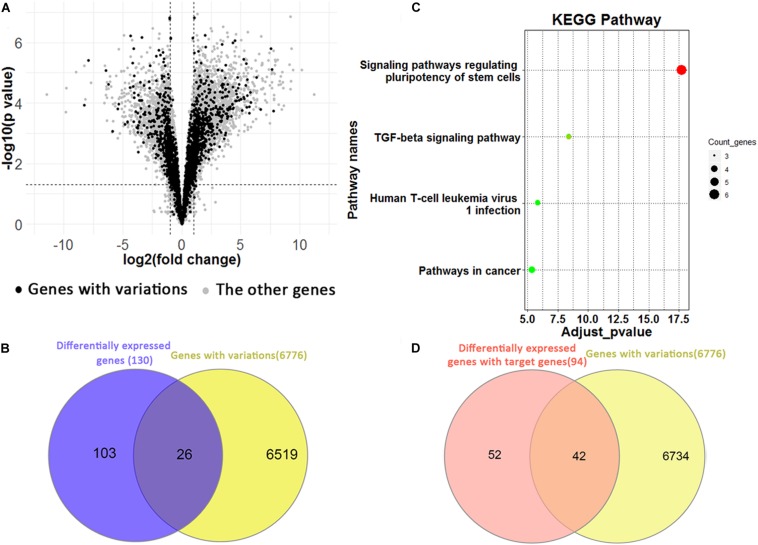
**(A)** Volcano plotting shows the differentially expressed genes between human ESC and BMP4-induced trophoblast cell lines. The black points are for the genes with a genomic variation, while the gray points are for the other genes. **(B)** The Venn plotting shows the genes both with a genomic variation and those exhibiting a differential expression from H1 to H1_BMP4. **(C)** The significant KEGG pathway for 26 genes. **(D)** The Venn plotting shows the genes both containing variations, and 94 differentially expressed target genes, which were regulated by downstream signaling proteins of BMP4.

We identified 5,688 differentially expressed genes between H1 and H1_BMP4 (FDR p-value ≤ 0.05 and fold change ≥ 2 or ≤1/2) (Figure 5A). In the genes, there are 3,763 up-regulated and 1,925 down-regulated genes. Since each genomic variation was assigned to a gene, we could identify the genomic variations that associate differentially expressed genes.

One-hundred-and-twenty-nine differential expression genes were determined using stricter criteria (*p*-value ≤ 1E–5 and fold change ≥ 4 or ≤1/4). We found a total of 26 genes with variations in the differentiation process out of the 129 differential expression genes (Figure 5B). The functional annotation tool of KEGG was applied to the 26 genes (Figure 5C). As expected, two pathways implicated in regulating human ESC differentiation was found with an adjusted *p*-value < 0.05, namely the “signaling pathways regulating pluripotency of stem cells” and the “TGF-beta signaling pathway,” which represent the differentiation pathways. For instance, inhibition of the TGF-beta signaling pathway could be sufficient for the derivation and long term expansion of human trophoblast cells and the placenta (Xu et al., 2017; Knöfler et al., 2019).

In the induction by BMP4, BMP ligands are bound to the BMP4 complex, then transphosphorylate the intracellular signaling proteins, Smad1/5/8. The phospho-Smad1/5/8 interacts with Smad4, and the complex translocates into the nucleus where it interacts with transcription cofactors and regulates expression of target genes in a cell type-specific manner (Shimasaki et al., 2004). Based on these biological processes, 235 target genes of Smad 1/4/5/6 were retrieved from the TF targets in the Harmonizome database (Rouillard et al., 2016). Among them, 94 are differentially expressed between H1 and H1_BMP4 (FDR *p*-value ≤ 0.05 and fold change ≥ 2 or ≤1/2). There are 72 up-regulated (30.64%) and 22 down-regulated genes (9.36%). In the 94 BMP4-induced genes, 42 (44.68%) contain the genomic variations (Figure 5D), suggesting a tight association between the variation and BMP4 induced differentiation.

In particular, by excluding the variations associating the epigenetic modification alteration, six genomic variations, which associate differentially expressed genes, were identified (Table 3, and Supplementary Excel File S3). Five of the six variations were located in the promoter regions of genes MEF2C, TNFAIP8, TEX2, INHBA-AS1, and CAMK2N1. The remaining one was at the enhancer of gene COL1A2.

**TABLE 3:** Information of six genomic variations.

Chr	Start	Ref	Alt	Location	Gene.refGene	Modification types
chr5	118605129	ATG	A	Intronic	TNFAIP8	H3K4me3
chr5	88179358	A	G	ncRNA_intronic	MEF2C	H3K4me3
chr17	62339443	G	T	Intronic	TEX2	H3K4me3
chr7	41740828	G	A	ncRNA_intronic	INHBA-AS1	H3K4me3
chr1	20811135	T	C	Intronic	CAMK2N1	H3K4me3
chr7	94023911	A	G	UTR5	COL1A2	H3K27ac

*Column Location lists the genomic region of the variation. ncRNA_intronic means non-coding RNA genes. Gene.refGene is the gene associating the variation. Modification types indicate the H3K4me3 or H3K27ac around the variation in both H1 and H1_BMP4.*

We highlighted a core function of the six genes (MEF2C, TNFAIP8, TEX2, INHBA-AS1, CAMK2N1, and COL1A2) in differentiation and placenta development. TNFAIP8 plays a role in immune homeostasis, inflammatory responses, tumor genesis, and development. TNFAIP8 is also highly expressed in most normal human tissues especially for immunity-related tissues like the placenta (Zhang et al., 2018). INHBA encodes a member of the TGF-β (transforming growth factor-beta) superfamily of proteins which has been proven to promote the differentiation of human embryonic stem cells into trophoblasts (Pucéat, 2007). COL1A2 encodes one of the chains for type I collagen, the fibrillar collagen found in most connective tissues, and it is an early stage marker of osteoblast differentiation (Parisuthiman et al., 2005). MEF2C plays a pivotal role in myogenesis, neural crest, and craniofacial development, and may have an influence on maintaining the differentiated state of muscle cells (Zweier et al., 2010). In a recent study, dysregulation of MEF2 expression or signaling in early pregnancy may be associated with placenta-related pregnancy disorders, including preeclampsia (Li et al., 2017). In total, the six genes both associate a genomic variation in their regulatory regions and show differential expression from H1 to H1_BMP4, suggesting that the genomic variations associated with differentiation.

Since the six genomic variations are in non-coding regions, we assessed the effect of the variation in altering the binding affinity of TFs. We applied JASPAR2018^3^ to calculate the motif score of 12-bp DNA sequences around the six SNVs/indels to the TFs that bind at those variation-harbored regions (Khan et al., 2018). We listed the motif scores in Supplementary Excel File S3. We found that the allele G of a variation (chr5: 88179358 A > G), which is at the enhancer of gene MEF2C, can increase the motif scores ≥ 10 in the induced cells (Table 4). it should be considered, however, that the MEF2C expression level is ∼4 fold higher in H1_BMP4 than in H1. The results mean that the genomic variation accounts for the increase of MEF2C expression by increasing TFs’ affinity at MEF2C’s promoter. Moreover, the H2K27ac does not show a significant change between the two cell lines. We confirmed that the MEF2C expression increase is caused by the genomic variation.

**TABLE 4:** The TF motif scores of MEF2C.

TF_Name	Start	End	Strand	H1 Expression(TPM)	H1_BMP4 Expression (TPM)	H1_BMP4_motif score	H1_motif score	motif score_diff
KLF16	11	21	–	14	15.52	14.81	10.90	3.90
ZNF740	7	16	–	5.4	7.29	12.63	7.09	5.54
ZNF740	6	15	–	5.4	7.29	10.66	5.53	5.13
NR2C2	10	24	+	9.27	12.99	10.09	3.87	6.22

*The TFs in this table were chosen as follows: (1) the motif scores of the TFs larger than 10 in H1 or H1_BMP4; (2) TPM value of the TF larger than 3 in both H1 and H1_BMP4; (3) the fold change of the expressed TF genes between H1 and H1_BMP4 less than 2.*

**FIGURE 6:**
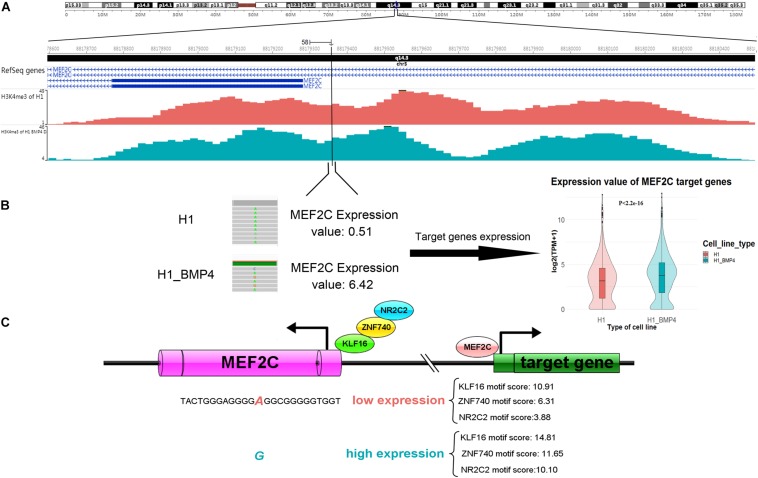
A genomic variation (chromosomes 5: 88179358 A > G) inserts its function in differentiation by affecting MEF2C’ expression, by altering the affinity of TFs binding. **(A)** The H3K4me3 track of H1 and H1_BMP4 around the variation site. **(B)** The gene expression of TF MEF2C was significantly increased (left panel). Violin plotting (right panel) shows that the gene expression of target genes of TF ‘MEF2C’ was significantly up-regulated between H1 and H1_BMP4 (*p* < 2.2E-16). **(C)** During differentiation, increased expression levels of the MEF2C gene is due to a genomic alteration, chromosomes 5: 88179358 A > G, which is at a binding site of TFs KLF16, NR2C2, and ZNF740. Allele G shows a higher affinity to TFs in the induced cell. A high expression of MEF2C leads to an increased expression of TF MEF2C’s target genes, subsequently affecting the differentiation.

Importantly, the MEF2C gene itself encodes a TF protein. More recently, MEF2 was proven to regulate human trophoblast differentiation and invasion (Li et al., 2017). We retrieved the list of 954 MEF2C target genes reported in ENCODE TF targets (Rouillard et al., 2016). We then compared the TF MEF2C target genes’ expression between H1 and H1_BMP4. The result showed that they are significantly up-regulated in the transition from human ESC and the trophoblast (paired sample *t*-test, *p* < 2.2E-16) (Figure 6).

Functional annotations by Enrichr (Kuleshov et al., 2016) on KEGG (Kyoto Encyclopedia of Genes and Genomes) and GO (Gene ontology) suggest the target genes of TF MEF2C were enriched in the “NF-kappa B signaling pathway” (Pollheimer and Knöfler, 2005), “Primary immunodeficiency,” “Systemic lupus erythematosus,” “positive regulation of NF-kappaB transcription factor activity,” “positive regulation of immune response,” and so on (adjust *p*-value ≤ 0.05, Supplementary Figure S5). Literature suggests that the placenta is an important immune-related tissue (Erkers et al., 2017). We therefore considered that the target genes of TF MEF2C have a significant impact on the cell differentiation process.

Briefly, the gene expression of MEF2C was significantly increased due to the high-affinity binding sites of TFs like KLF16, NR2C2, and ZNF740, a variation site located in MEF2C (chromosomes 5: 88179358 A > G). Further, the expression levels of these three TFs were not significantly different between H1 and H1_BMP4, suggesting that MEF2C’s expression increase is not caused by a rise in the abundance of the TFs of MEF2C, but due to the genomic variation (Figure 6). Interestingly, literature indicates that simultaneous depletion of KLF2, KLF4, and KLF5 leads to differentiation of the embryonic stem cell, and it has been postulated that other members of the KLF family, such as KLF16, may play similar roles in embryonic stem cells (Jiang et al., 2008; Andreoli et al., 2010). It has been established that NR2C2 (TR4) plays a critical role in embryonic development and differentiation.

NR2C2 is expressed in blastocysts and embryonic stem cells and can act as transcriptional activators in hESC (Shyr et al., 2009; O’Geen et al., 2010). Altogether, this indicates that due to the up-regulation of MEF2C, its target genes were up-regulated. Importantly, the upregulation of MEF2C is caused by the genomic variation (chr5: 88179358 A > G), which alters the affinity of MEF2C’s TFs.

## Discussion

Understanding the genetic underpinnings of complex traits remains a major challenge in human genetics. In this study, we obtained paired genomic DNA sequences of human ESC and the trophoblast from three cell lines (H1, H9, and MRucR) through whole genome sequencing, and integrated the epigenomic (DNA methylation, histone modifications and chromatin accessibility) and transcriptomic datasets to investigate the impact of the genome variations in human ESC differentiation to the trophoblast. We found that the SNVs and indels generally tend to be located in the intron regions rather than in the other regions.

We focused on the gene expression variation caused by genomic variation rather than the epigenetic variation. Six genomic variations were identified. One of them, located in MEF2C (chromosomes 5: 88179358 A > G), is a TF. This SNV increased the TF binding strength to regulate gene expression directly. Thereby, leading to an increase in the expression of downstream target genes affecting the differentiation of human ESC into the trophoblast. It suggested that the variations in the non-coding region played an important role in the differentiation process.

The inducer, BMP4, is the most significant factor to differentiate to the trophoblast. BMP4 is able to inhibit the Activin/Nodal signaling pathway and activate the BMP signaling pathway, which is required for human ESCs to differentiate into trophoblasts (Xu et al., 2002). We showed that ∼45% (42 genes) of the differentially expressed BMP4-induced genes associated with genomic variations. Although genomic variations are not possible to be the only dominant factor in differentiation, some genomic variations indeed have an effect on differentiation. There are two limitations in this work. The first is that the conclusion is confined to only three pairs of cell lines. A similar analysis should be carried out in more cell lines of iPSC. The second limitation is that comprehensive biochemical experiments are still needed to validate the conclusion. CRISPR technology in cultured cells could be employed to prove the role of genomic variation in the differentiation process (Zhou et al., 2014, p. 2).

## Data Availability Statement

The raw sequence data reported in this paper have been deposited in the Genome Sequence Archive (Wang et al., 2017) at the BIG Data Center (Zhang et al., 2019), Beijing Institute of Genomics (BIG), Chinese Academy of Sciences, under accession number CRA002190, which is publicly accessible at https://bigd.big.ac. cn/gsa.

## Author Contributions

All authors contributed to the study conception and design, analyzed and discussed the results. XS conceived the project and completed the core program. HaL, HoL, and YL performed the computational analysis. HaL and HoL wrote the manuscript.

## Conflict of Interest

The authors declare that the research was conducted in the absence of any commercial or financial relationships that could be construed as a potential conflict of interest.

## Footnotes

1 http://broadinstitute.github.io/picard

2 https://www.encodeproject.org/

3 http://jaspar.genereg.net/
