# Corrigendum: Effect for Human Genomic Variation During the BMP4-Induced Conversion From Pluripotent Stem Cells to Trophoblast

**DOI:** 10.3389/fgene.2020.00538

**Published:** 2020-05-28

**Authors:** Hai-tao Li, Yajun Liu, Hongde Liu, Xiao Sun

**Affiliations:** ^1^State Key Laboratory of Bioelectronics, School of Biological Science and Medical Engineering, Southeast University, Nanjing, China; ^2^The Second Affiliated Hospital of Zhengzhou University, Zhengzhou, China; ^3^Academy of Medical Sciences of Zhengzhou University Translational Medicine Platform, Zhengzhou University, Zhengzhou, China

**Keywords:** genomic variations, pluripotent stem cells, trophoblast, whole genome sequencing, epigenomic and transcriptomic data

In the original article, there was an error in the Acknowledgments. It was written that Professor Toshihiko Ezashi and Professor R. Michael Roberts provided “three human pluripotent stem cells”; however, this is not accurate. They provided “genomic materials obtained from three human pluripotent stem cells.”

The authors apologize for this error and state that this does not change the scientific conclusions of the article in any way. The original article has been updated.

