# Supplementary material for: Effect for Human Genomic Variation During the BMP4-Induced Conversion From Pluripotent Stem Cells to Trophoblast
Source: Front Genet. 2020 Apr 7;11:230. doi: 10.3389/fgene.2020.00230 (PMC7154154; doi:10.3389/fgene.2020.00230)
Supplement: Supplementary file 4 [file Presentation_1.pdf]

Supplementary Material

Effect for human genomic variation during the BMP4-induced conversion from pluripotent stem cells to trophoblast

Hai-tao Li[1†], Yajun Liu[2,1†], Hongde Liu[1*], Xiao Sun[1*]

[1]State Key Laboratory of Bioelectronics, School of Biological Science and Medical Engineering, Southeast University, Nanjing, 210096, P.R. China

[2] The Second Affiliated Hospital of Zhengzhou University, No. 2 Jingba road, Zhengzhou; Academy of Medical Sciences of Zhengzhou University Translational Medicine platform, Zhengzhou University, No.100 Science Avenue, Zhengzhou City, Henan Province, 450001, P.R. China.

*** Correspondence:**
Hongde Liu; liuhongde@seu.edu.cn

Xiao Sun; xsun@seu.edu.cn

[†]These authors contributed to the paper as first authors.

Supplementary Figures and Tables

1.1 Supplementary Table

Table S1. The information of cell lines analyzed in this study

SampleID	Sex	Cell line type	Normal/Patient
**H1**	Male	hESC	Normal
**H1_BMP4**	Male	trophoblast	Normal
**H9**	Female	hESC	Normal
**H9_BMP4**	Female	trophoblast	Normal
**MRucR**	Female	iPSC	Patient (preeclampsia)
**MRucR_BMP4**	Female	trophoblast	Patient (preeclampsia)

Table S2. The bioinformatics tools used in this study

**Analysis**	**Software**	**Notes**	**Version**
Quality control	In house		
Alignment	BWA	Map the sequencing reads to the reference genome and the BAM file was obtained	0.7.8-r455
	SAMtools	Sort bam	1
	Picard	Merge the bam file from the same sample and mark the duplicate reads	1.111
SNP/InDel detection	Strelka2	Detect and filter SNV and InDels	2
	ANNOVAR	Annotate variation site	2018Apr16
SV detection	BreakDancer	Detect SV	V0.0.1
	ANNOVAR	Annotate variation site	2018Apr16

Table S3. The data sets from different laboratories analyzed cell lines of H1 and BMP4-induced trophoblast H1_BMP4 in this paper. All of the data can be retrieved through the corresponding GEO number.

**Data**		**H1 (GSE16256; GSE18927)**	**H1_BMP4 (GSE16256; GSE18927)**
DNA methylation	WGBS(Whole-Genome Bisulfite Sequencing)	GSM429321	GSM602254
		GSM429322	GSM602255
Histone	H3K27me3	GSM605308	GSM896164

Modifications ChIP-Seq		GSM466734	GSM753431
	H3K27ac	GSM466732	GSM864800
		GSM663427	GSM753427
	H3K4me3	GSM605315	GSM753441
		GSM469971	GSM753442
Chromatin accessibility	DNase-Seq	GSM878616	GSM878628
		GSM878628	GSM878614
Gene expression	RNA-Seq	GSM915328	GSM915320
		GSM915329	GSM915321

Table S4. Overview of data production quality

**Sample name**	**Novo ID**	**Raw reads**	**Raw data(G)**	**Raw depth(x)**	**Effective(%)**	**Error(%)**	**Q20(%)**	**Q30(%)**	**GC(%)**
**H1**	D17041657	3.32E+08	99.64	34.43	99.81	.04	92.92	84.66	41.55
**H1_BMP4**	D17041658	3.19E+08	95.7	33.06	99.8	0.02	95.81	90.17	41.44
**H9**	D17041659	3.40E+08	102.06	35.26	99.83	0.02	95.97	90.42	41.16
**H9_BMP4**	D17041660	3.46E+08	103.71	35.83	99.79	0.02	95.8	90.16	41.17
**MRucR**	D17041661	3.08E+08	92.54	31.97	99.82	0.03	92.89	84.54	41.25

**MRucR_BMP4**	D17041662P	3.03E+08	91.02	31.45	99.73	0.04	92.84	84.54	41.23

Note:

(1) Sample name: Sample name.

(2) Novo ID: Novogene ID of the sample.

(3) Lane: The flowcell ID and lane number during the sequencing (FlowcellID_LaneNumber).

(4) Raw reads: The number of sequencing reads pairs; four lines will be considered as one unit according to FASTQ format.

(5) Raw data (G): The original sequence data volume.

(6) Raw depth (x): The original sequence depth.

(7) Effective (%): The percentage of clean reads in all raw reads.

(8) Error (%): The average error rate of all bases.

(9) Q20: The percentage of bases with Phred score ≥20.

(10) Q30: The percentage of bases with Phred score ≥30.

(11) GC: The percentage of G and C in the total bases.

Table S5. Mapping rate and coverage

**Sample:1**	**H1**	**H1_BMP4**	**H9**	**H9_BMP4**	**MRucR**	**MRucR_BMP4**
**Total:2**	662994998 (100%)	636669960 (100%)	679231148 (100%)	689959414 (100%)	615828596 (100%)	605144646 (100%)
**Duplicate:3**	75417833 (11.40%)	88802963 (13.96%)	92083713 (13.57%)	94334955 (13.68%)	72603333 (11.82%)	60969448 (10.13%)
**Mapped:4**	661426654	636144377	678740317 (99.93	689360772 (99.91%)	614252737	601732042 (99.44%)

	(99.76%)	(99.92%)	%)		(99.74%)	
**Properly mapped:5**	646497086 (97.51%)	623844710 (97.99%)	663535732 (97.69%)	675030636 (97.84%)	601012644 (97.59%)	584154878 (96.53%)
**PE mapped:6**	660208754 (99.58%)	635643528 (99.84%)	678266476 (99.86%)	688779994 (99.83%)	613047432 (99.55%)	598995418 (98.98%)
**SE mapped:7**	2435800 (0.37%)	1001698 (0.16%)	947682 (0.14%)	1161556 (0.17%)	2410610 (0.39%)	5473248 (0.90%)
**With mate mapped to a different chr:8**	9829294 (1.48%)	7618776 (1.20%)	10883966 (1.60%)	9300750 (1.35%)	8687356 (1.41%)	10980468 (1.81%)
**With mate mapped to a different chr ((mapQ>=5)):9**	8435837 (1.27%)	6389141 (1.00%)	9543489 (1.41%)	7950739 (1.15%)	7513706 (1.22%)	9505529 (1.57%)
**Average_sequencing_depth:10**	33.27	32.13	34.38	34.84	31.05	30.21
**Coverage:11**	99.84%	99.85%	99.14%	99.14%	99.11%	99.11%
**Coverage_at_least_4X:12**	99.71%	99.71%	98.97%	98.98%	98.94%	98.94%
**Coverage_at_least_10X:13**	98.90%	98.81%	98.65%	98.69%	98.52%	98.51%
**Coverage_at_least_20X:**	91.42%	90.66%	95.35%	96.10%	92.29%	91.60%

**14**						

Note:

(1) Total: The total number of clean reads

(2) Duplicate: The number of duplication reads

(3) Mapped: The number of total reads that mapped to the reference genome (percentage)

(4) Properly mapped: The number of reads that mapped to the reference genome and the direction is right

(5) PE mapped: The number of pair-end reads that mapped to the reference genome (percentage)

(6) SE mapped: The number of single-end reads that mapped to the reference genome

(7) With mate mapped to a different chr: The number of mate reads that mapped to the different chromosomes (percentage)

(8) With mate mapped to a different chr (mapQ>=5): The number of mate reads that mapped to the different chromosomes and the MAQ >5

(9) Average_sequencing_depth: The average sequencing depth that mapped to the reference genome

(10) Coverage: The sequence coverage of genome

(11) Coverage_at_least_4X: The percentage of bases with depth >4× in whole genome bases

(12) Coverage_at_least_10X: The percentage of bases with depth >10× in whole genome bases

(13) Coverage_at_least_20X: The percentage of bases with depth >20× in whole genome bases

Table S6. The top 20 key TFs were selected around the variations using HOMER in H1.

**Rank**	**TF**	**Description**	**Count**	**MotifScore**	**Reference**
**1**	SREBP1A	Sterol regulatory element-binding protein 1	314	10.71	(Lecomte et al., 2010)
**2**	SREBP2	Sterol Response Element Binding Protein 2	262	11.31	
**3**	GATA2	GATA-binding factor 2	213	10.65	(Ray et al., 2009; Bai et

					al., 2012)
**4**	OCT4	Octamer-binding transcription factor 4	186	10.77	(Wang et al., 2012)
**5**	STAT3	Signal transducer and activator of transcription 3	173	10.39	(Poehlmann et al., 2005)
**6**	NFAT	Nuclear factor of activated T-cells	166	10.58	(Li et al., 2011)
**7**	KLF3	Kruppel-like factor 3	155	10.54	(Bruce et al., 2007)
**8**	NR5A2	Nuclear receptor subfamily 5, also known as the liver receptor homolog-1	152	10.58	(Heng et al., 2010)
**9**	TEAD1	Transcriptional enhancer factor TEF-1	136	10.58	(Nishioka et al., 2008)
**10**	IRF3	interferon regulatory factor-3	128	10.63	
**11**	TEAD2	TEA Domain Transcription Factor 2	127	10.56	(Nishioka et al., 2008)
**12**	P63	Tumor Protein p63	116	11.26	(Li et al., 2014)
**13**	STAT1	Signal transducer and activator of transcription 1	116	10.69	(Pereira de Sousa et al., 2017)
**14**	FOXP1	Forkhead box protein P1	115	10.34	
**15**	TEAD3	Transcriptional Enhancer Factor TEF-3	102	10.77	(Jacquemin et al., 1998)
**16**	KLF10	Kruppel-like factor 10	98	10.81	
**17**	AP-1	Activator protein 1	97	11.16	(Kubota et

					al., 2015)
**18**	ZNF322	Zinc Finger Protein 322	91	10.80	
**19**	OCT6	Octamer-binding transcription factor 6	90	10.57	(Knöfler et al., 2019)
**20**	SOX4	Sex Determining Region Y (SRY)-Box 4	88	10.49	

Table S7. The top 20 key TFs were selected around the variations using HOMER in H1_BMP4.

**Rank**	**TF**	**Description**	**Count**	**MotifScore**	**Reference**
**1**	SREBP1A	Sterol regulatory element-binding protein 1	312	10.71	(Lecomte et al., 2010)
**2**	SREBP2	Sterol Response Element Binding Protein 2	262	11.31	
**3**	GATA2	GATA-binding factor 2	212	10.65	(Ray et al., 2009; Bai et al., 2012)
**4**	STAT3	Signal transducer and activator of transcription 3	176	10.39	(Poehlmann et al., 2005)
**5**	NFAT	Nuclear factor of activated T-cells	163	10.58	(Li et al., 2011)
**6**	CEBPA	CCAAT Enhancer Binding Protein Alpha	147	10.81	(Simmons et al., 2008)
**7**	GATA3	GATA binding protein 3	145	11.15	(Kubaczka et al., 2015)
**8**	TEAD	Transcriptional enhancer factor TEF-	134	10.58	(Nishioka et al.,

	1	1			2008)
**9**	KLF4	Kruppel-like factor 4	132	11.37	(Abad et al., 2013)
**10**	KLF3	Kruppel-like factor 3	130	10.54	(Bruce et al., 2007)
**11**	TEAD2	TEA Domain Transcription Factor 2	127	10.56	(Nishioka et al., 2008)
**12**	IRF3	interferon regulatory factor-3	126	10.63	
**13**	THRB	Thyroid hormone receptor beta	117	10.66	
**14**	P63	Tumor Protein p63	116	11.26	(Li et al., 2014)
**15**	STAT1	Signal transducer and activator of transcription 1	116	10.69	(Pereira de Sousa et al., 2017)
**16**	FOXP1	Forkhead box protein P1	115	10.34	
**17**	ZEB2	Zinc finger E-box binding homeobox 2	109	10.43	(DaSilva-Arnold et al., 2019)
**18**	TEAD3	Transcriptional Enhancer Factor TEF-3	102	10.77	(Jacquemin et al., 1998)
**19**	KLF10	Kruppel-like factor 10	100	10.82	
**20**	AP-2 α	Adaptor Protein complex 2, α subunit	96	11.17	(Biadasiewicz et al., 2011)

Table S8. The key TFs were selected around the variations using HOMER between H1 and H1_BMP4

TF	Description	Motif count difference (H1-H1_BMP4)	Motif count in H1	Motif count in H1_BMP4	Reference
Zfp281	Krüppel‐like zinc finger TF	39	47	8	(Ishiuchl et al., 2019)
OCT	Octamer-binding proteins	30	30	0	(Xu et al., 2002)
KLF3	Kruppel-like factor 3	17	18	1	(Bruce et al., 2007)

1.2 Supplementary Figures

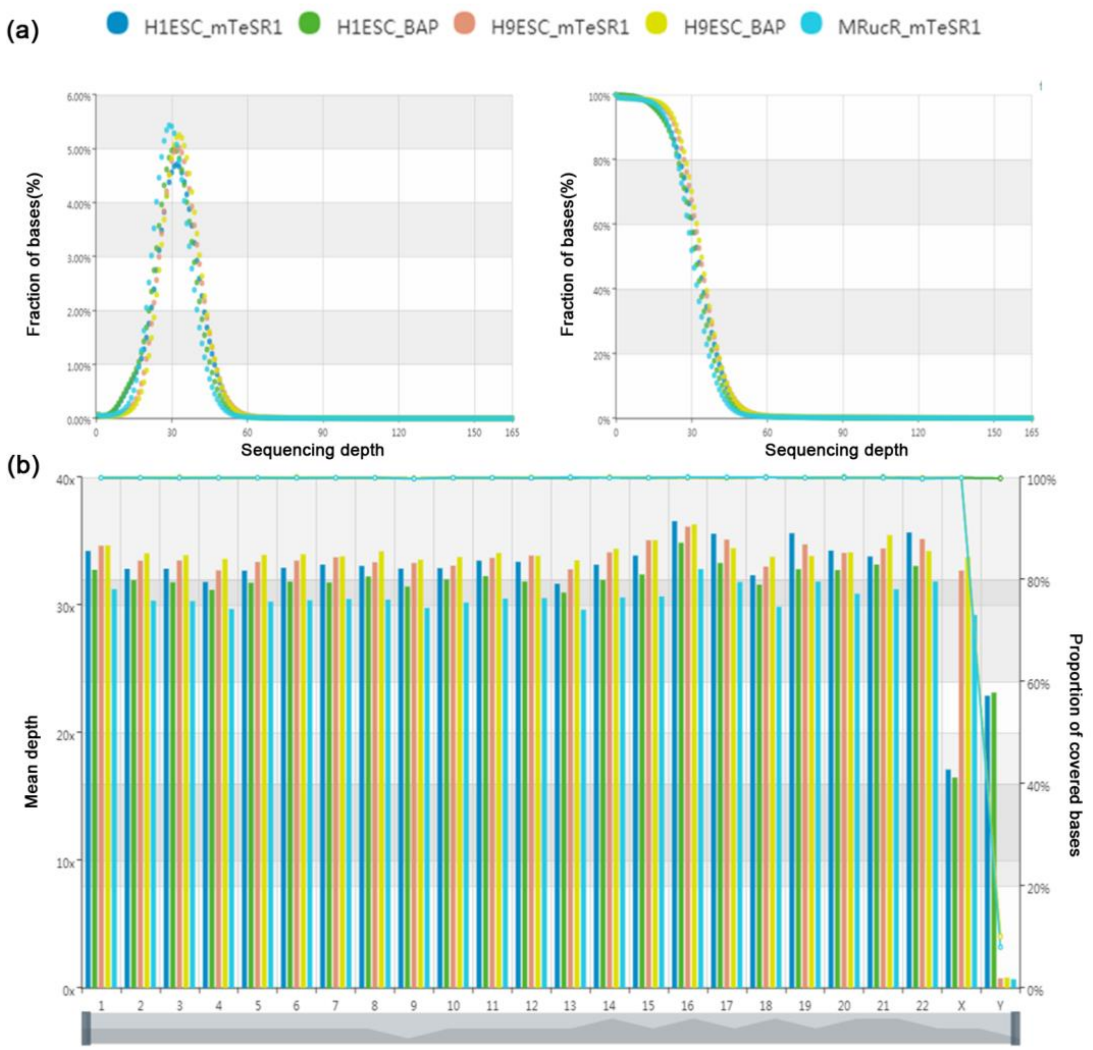

Figure S1 Sequencing Depth and Coverage Distribution. (a) Sequencing depth. The left figure is the ratio of bases with different sequencing depth. The x-axis is sequencing depth; the y-axis is the fraction of bases with the given sequencing depth. The right figure is accumulative base ratio with different depth. The x-axis is sequencing depth; the y-axis is the fraction of bases above the given sequencing depth. For example, the sequencing depth of 0× corresponds to the base ratio of 100%, showing that 100% base's sequencing depth >0×. (b) The coverage depth (the left coordinate) and coverage rate (the right coordinate) of chromosome. The x-axis is chromosome number; the left y-axis is the average depth of each chromosome (Raw data/length_of_chromosome); the right y-axis is the fraction of covered on each chromosome (The number of bases covered by reads/total number of bases).

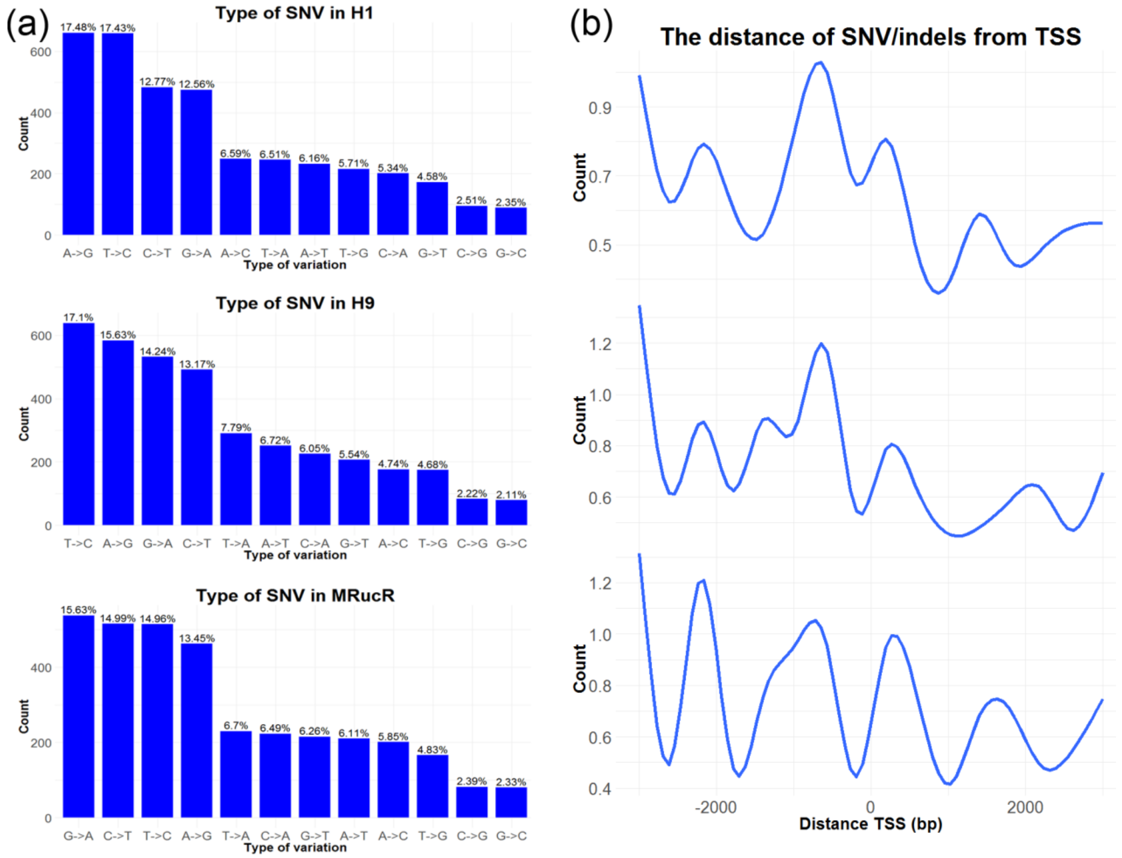

Figure S2. General features of paired whole genome sequencing genomic data of hESC and trophoblast from three cell lines (H1, H9 and MRucR). From top to bottom were matched hESC-trophoblast cell lines of H1, H9 and MRucR, respectively. (a) Frequency of SNV single-base changes between hESC and trophoblast cell lines. (b) The distribution of SNV/indels distance from 2500bp upstream and downstream of TSSs.

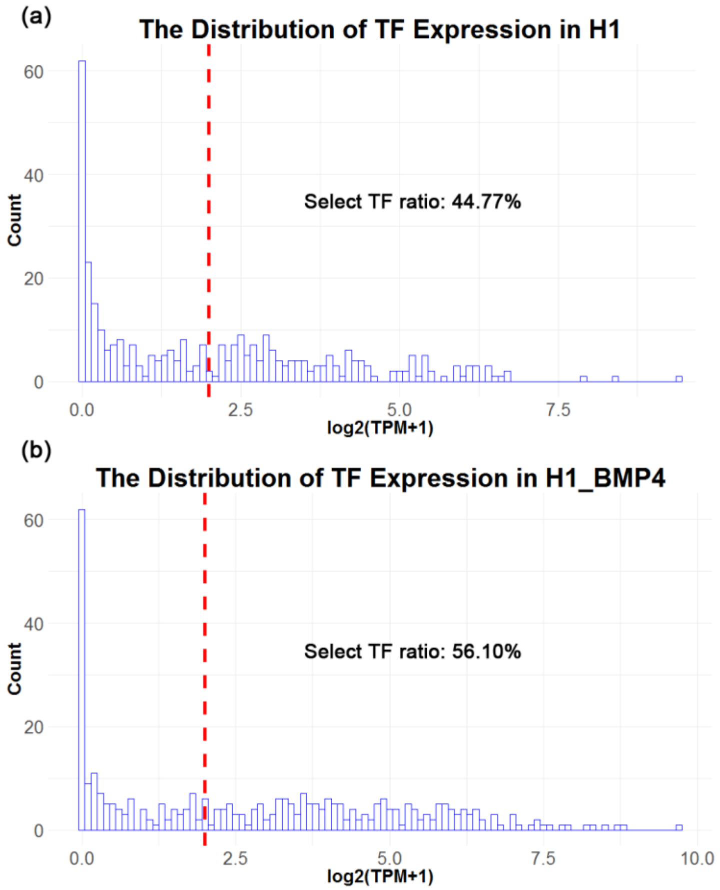

Figure S3. The distribution of TF expression in H1 (a) and H1_BMP4 (b).

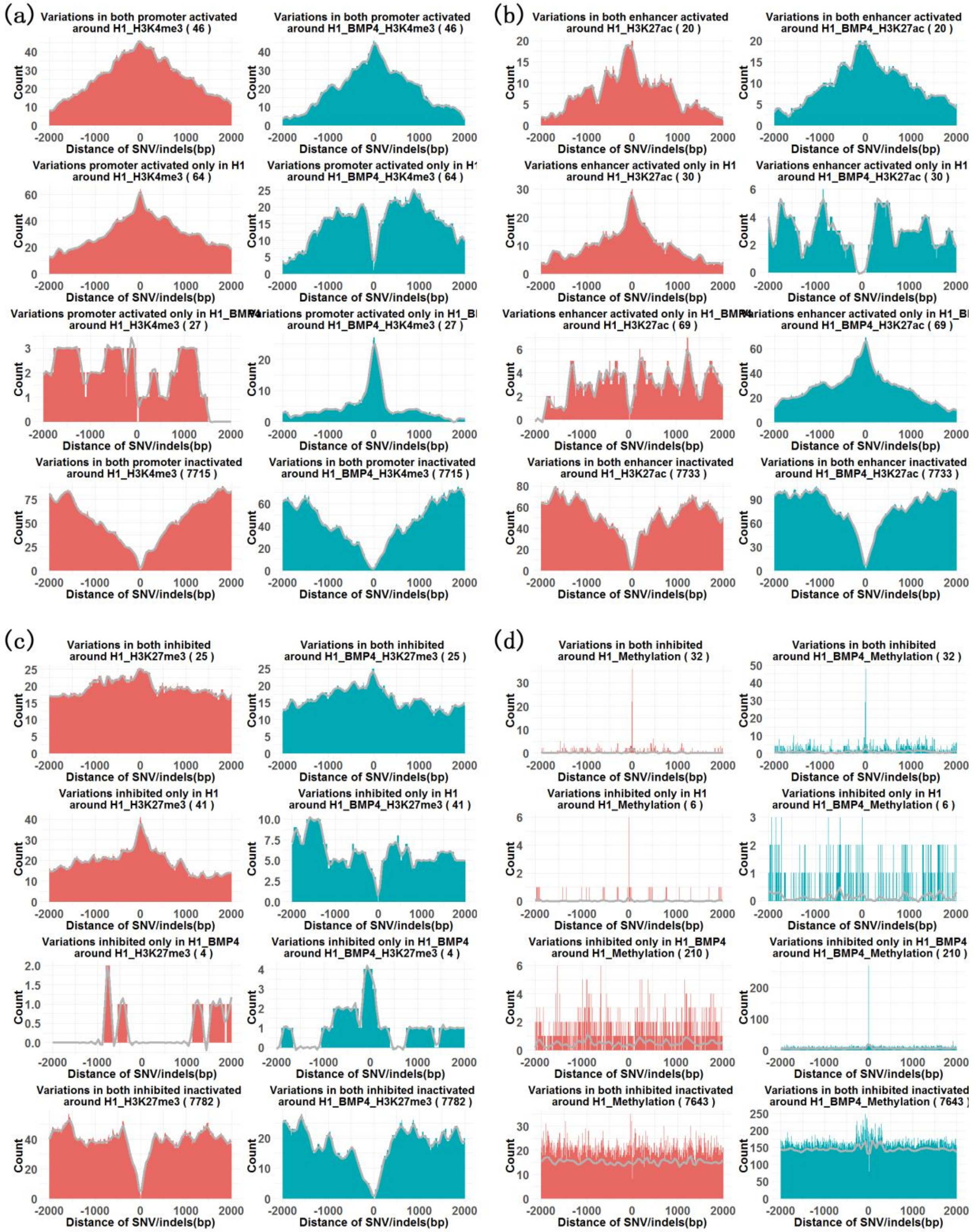

Figure S4. The peaks surrounding SNV/indels of histone modifications and DNA methylation were counted. X-axis is the upstream and downstream 2Kbp sequence of SNV/indels. Y-axis is the number of peak counts. Subplots of histone modifications and DNA methylation are (a) H3K4me3, (b) H3K27ac, (c) H3K27me3 and (d) DNA methylation, respectively. The left column of each subplot is

peak counts in H1, while the right column of each subplot is that of H1_BMP4. The first line of each subplot is the variation located in the peak region of the epigenetic data of H1 and the peak region of the epigenetic data of H1_BMP4. The second line of each subplot is the variation located in the peak region of the epigenetic data of H1 but not in the peak region of the epigenetic data of H1_BMP4. The third line of each subplot is the variation didn't locate in the peak region of the epigenetic data of H1 but the peak region of the epigenetic data of H1_BMP4. The fourth line of each subplot is the variation didn't locate in the peak region of the epigenetic data of H1 and not in the peak region of the epigenetic data of H1_BMP4.

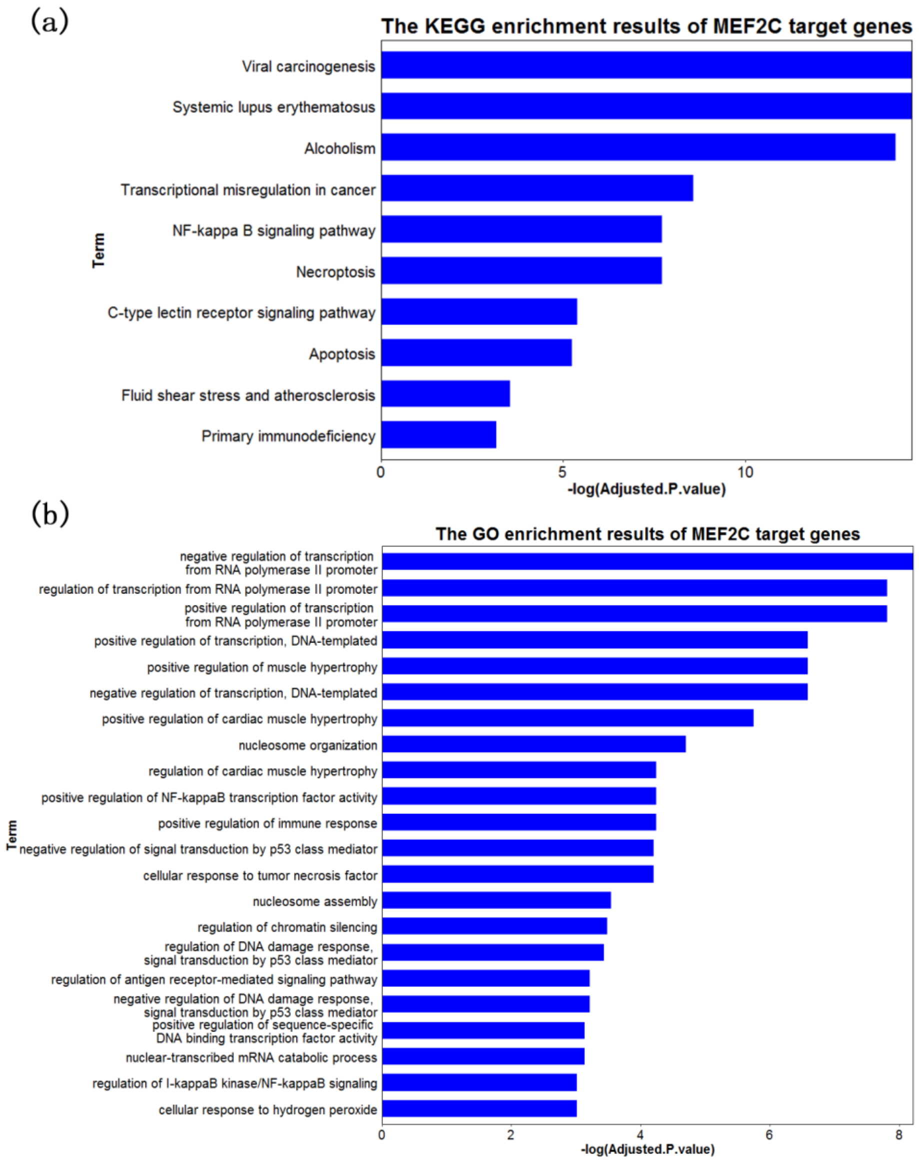

Figure S5. The significantly pathways and biological process for target genes of MEF2C transcription factors use Enrichr. An adjust P-vaule, 0.05, was chosen as significant thresholds upon filtering the pathway data.

Reference

Abad, M., Mosteiro, L., Pantoja, C., Cañamero, M., Rayon, T., Ors, I., et al. (2013). Reprogramming *in vivo* produces teratomas and iPS cells with totipotency features. *Nature* 502, 340–345. doi:10.1038/nature12586.

Bai, Q., Assou, S., Haouzi, D., Ramirez, J.-M., Monzo, C., Becker, F., et al. (2012). Dissecting the first transcriptional divergence during human embryonic development. *Stem Cell Rev Rep* 8, 150–162. doi:10.1007/s12015-011-9301-3.

Biadasiewicz, K., Sonderegger, S., Haslinger, P., Haider, S., Saleh, L., Fiala, C., et al. (2011). Transcription factor AP-2α promotes EGF-dependent invasion of human trophoblast. *Endocrinology* 152, 1458–1469. doi:10.1210/en.2010-0936.

Bruce, S. J., Gardiner, B. B., Burke, L. J., Gongora, M. M., Grimmond, S. M., and Perkins, A. C. (2007). Dynamic transcription programs during ES cell differentiation towards mesoderm in serum versus serum-freeBMP4 culture. *BMC Genomics* 8, 365. doi:10.1186/1471-2164-8-365.

DaSilva-Arnold, S. C., Kuo, C.-Y., Davra, V., Remache, Y., Kim, P. C. W., Fisher, J. P., et al. (2019). ZEB2, a master regulator of the epithelial-mesenchymal transition, mediates trophoblast differentiation. *Mol. Hum. Reprod.* 25, 61–75. doi:10.1093/molehr/gay053.

Heng, J.-C. D., Feng, B., Han, J., Jiang, J., Kraus, P., Ng, J.-H., et al. (2010). The Nuclear Receptor Nr5a2 Can Replace Oct4 in the Reprogramming of Murine Somatic Cells to Pluripotent Cells. *Cell Stem Cell* 6, 167–174. doi:10.1016/j.stem.2009.12.009.

Ishiuchi, T., Ohishi, H., Sato, T., Kamimura, S., Yorino, M., Abe, S., and Sasaki, H. (2019). Zfp281 shapes the transcriptome of trophoblast stem cells and is essential for placental development. Cell reports, 27(6), 1742-1754.

Jacquemin, P., Sapin, V., Alsat, E., Evain-Brion, D., Dollé, P., and Davidson, I. (1998). Differential expression of the TEF family of transcription factors in the murine placenta and during differentiation of primary human trophoblasts in vitro. *Dev. Dyn.* 212, 423–436. doi:10.1002/(SICI)1097-0177(199807)212:3<423::AID-AJA10>3.0.CO;2-1.

Knöfler, M., Haider, S., Saleh, L., Pollheimer, J., Gamage, T. K. J. B., and James, J. (2019). Human placenta and trophoblast development: key molecular mechanisms and model systems. *Cell. Mol. Life Sci.* 76, 3479–3496. doi:10.1007/s00018-019-03104-6.

Kubaczka, C., Senner, C. E., Cierlitza, M., Araúzo-Bravo, M. J., Kuckenberg, P., Peitz, M., et al. (2015). Direct Induction of Trophoblast Stem Cells from Murine Fibroblasts. *Cell Stem Cell* 17, 557–568. doi:10.1016/j.stem.2015.08.005.

Kubota, K., Kent, L. N., Rumi, M. A. K., Roby, K. F., and Soares, M. J. (2015). Dynamic Regulation of AP-1 Transcriptional Complexes Directs Trophoblast Differentiation. *Molecular and Cellular Biology* 35, 3163–3177. doi:10.1128/MCB.00118-15.

Lecomte, V., Meugnier, E., Euthine, V., Durand, C., Freyssenet, D., Nemoz, G., et al. (2010). A New Role for Sterol Regulatory Element Binding Protein 1 Transcription Factors in the Regulation of Muscle Mass and Muscle Cell Differentiation. *Mol Cell Biol* 30, 1182–1198. doi:10.1128/MCB.00690-09.

Li, X., Zhu, L., Yang, A., Lin, J., Tang, F., Jin, S., et al. (2011). Calcineurin-NFAT Signaling Critically Regulates Early Lineage Specification in Mouse Embryonic Stem Cells and Embryos. *Cell Stem Cell* 8, 46–58. doi:10.1016/j.stem.2010.11.027.

Li, Y., Moretto-Zita, M., Leon-Garcia, S., and Parast, M. M. (2014). p63 inhibits extravillous trophoblast migration and maintains cells in a cytotrophoblast stem cell-like state. *Am. J. Pathol.* 184, 3332–3343. doi:10.1016/j.ajpath.2014.08.006.

Nishioka, N., Yamamoto, S., Kiyonari, H., Sato, H., Sawada, A., Ota, M., et al. (2008). Tead4 is required for specification of trophectoderm in pre-implantation mouse embryos. *Mechanisms of Development* 125, 270–283. doi:10.1016/j.mod.2007.11.002.

Pereira de Sousa, F. L., Chaiwangyen, W., Morales-Prieto, D. M., Ospina-Prieto, S., Weber, M., Photini, S. M., et al. (2017). Involvement of STAT1 in proliferation and invasiveness of trophoblastic cells. *Reproductive Biology* 17, 218–224. doi:10.1016/j.repbio.2017.05.005.

Poehlmann, T. G., Fitzgerald, J. S., Meissner, A., Wengenmayer, T., Schleussner, E., Friedrich, K., et al. (2005). Trophoblast invasion: tuning through LIF, signalling via Stat3. *Placenta* 26 Suppl A, S37-41. doi:10.1016/j.placenta.2005.01.007.

Ray, S., Dutta, D., Rumi, M. A. K., Kent, L. N., Soares, M. J., and Paul, S. (2009). Context-dependent function of regulatory elements and a switch in chromatin occupancy between GATA3 and GATA2 regulate Gata2 transcription during trophoblast differentiation. *J. Biol. Chem.* 284, 4978–4988. doi:10.1074/jbc.M807329200.

Simmons, D. G., Natale, D. R. C., Begay, V., Hughes, M., Leutz, A., and Cross, J. C. (2008). Early patterning of the chorion leads to the trilaminar trophoblast cell structure in the placental labyrinth. *Development* 135, 2083–2091. doi:10.1242/dev.020099.

Wang, Z., Oron, E., Nelson, B., Razis, S., and Ivanova, N. (2012). Distinct lineage specification roles for NANOG, OCT4, and SOX2 in human embryonic stem cells. *Cell Stem Cell* 10, 440–454. doi:10.1016/j.stem.2012.02.016.

Xu, R. H., Chen, X., Li, D. S., Li, R., Addicks, G. C., Glennon, C., and Thomson, J. A. (2002). BMP4 initiates human embryonic stem cell differentiation to trophoblast. Nature biotechnology, 20(12), 1261-1264.
